# Does perioperative chemotherapy improve survival in upper tract urothelial carcinoma? A population based analysis

**DOI:** 10.18632/oncotarget.24694

**Published:** 2018-04-10

**Authors:** Hanan Goldberg, Zachary Klaassen, Thenappan Chandrasekar, Rashid Sayyid, Girish S. Kulkarni, Robert J. Hamilton, Neil E. Fleshner

**Affiliations:** ^1^ Division of Urology, Department of Surgical Oncology, Princess Margaret Cancer Centre, University Health Network and the University of Toronto, Toronto, Ontario, Canada

**Keywords:** upper tract urothelial carcinoma, chemotherapy, cancer specific mortality, other cause mortality

## Abstract

**Objectives:**

To evaluate the utilization and outcomes of perioperative chemotherapy in non-metastatic UTUC patients over the past decade using a large national database.

**Methods:**

All patients aged 18 and older diagnosed with non-metastatic UTUC between 2004 and 2013 were identified within the Surveillance, Epidemiology and End Results (SEER) database. Relevant clinical data was collected and predictors of cancer specific mortality (CSM) and other cause mortality (OCM) were analyzed.

**Results:**

The total cohort included 8,762 patients. Of these, 1,402 (16%) patients received chemotherapy, including only 35% of high-risk patients (>pT2 or N1). Treated patients had higher CSM (21.3% vs. 13.1%, p<0.001). Predictors of chemotherapy utilization included residence in Midwest states, tumor located in the ureter, higher stage and grade. Predictors of CSM included older age, residence in southern states, receipt of chemotherapy (HR = 1.151, 95% CI: 1.003-1.32, p=0.044), higher stage and grade. OCM was predicted by male gender, older age, ureteral tumor, and higher stage. A subset analysis of patients younger than 65 showed similar predictors, while an analysis of high risk patients demonstrated that chemotherapy receipt did not predict CSM or OCM.

**Conclusions:**

In this large contemporary non-metastatic UTUC cohort, chemotherapy utilization was found to be quite infrequent, but increasing steadily. Perioperative chemotherapy had no effect on CSM in high-risk patients, while correlated to higher CSM in the younger patients.

## INTRODUCTION

Upper tract urothelial carcinoma (UTUC) is a relatively rare disease, accounting for less than 5% of all urothelial cancers (UC) and 5-10% of all renal tumors [1, 2]. The role of perioperative chemotherapy, whether adjuvant or neoadjuvant, remains poorly defined. However, utilization of chemotherapy in bladder UC patients has been studied extensively. Randomized controlled trials have demonstrated an overall survival (OS) benefit of cisplatin-based neoadjuvant chemotherapy for muscle-invasive bladder cancer [3–5]. Moreover, a meta-analysis of 11 randomized controlled trials showed a 5% and 9% improvement in OS and cancer specific survival (CSS), respectively, with neoadjuvant chemotherapy [6]. Similarly, although the evidence for adjuvant chemotherapy in bladder UC is less robust, several trials have exhibited a potential advantage in CSS with adjuvant cisplatin-based chemotherapy in high risk patients (>pT3 or pN1) [7–9].

Due to the rarity of UTUC, completion of large prospective studies examining the role of perioperative chemotherapy has been difficult to achieve. As a result, UTUC has been managed similarly to bladder UC, due to the presumption that it manifests a similar disease biology when compared stage for stage [10]. Subsequently, there has been an increasing trend of chemotherapy utilization in UTUC patients, based on the extrapolation of its benefit in bladder UC. Some studies have demonstrated measurable survival benefits with chemotherapy in metastatic or unresectable UTUC patients [11–13]. However, in non-metastatic UTUC patients, the benefit of perioperative chemotherapy in improving CSS and OS is still controversial. Nearly all available literature on chemotherapy utilization in UTUC was done in the adjuvant setting while only a minority examined its role in the neoadjuvant setting [14–16].

A recently published study looking at SEER-Medicare data assessed perioperative chemotherapy utilization in UTUC patients over the age of 65, showing no added benefit for either neoadjuvant or adjuvant chemotherapy [17]. The objective of the current study is to evaluate the utilization and trends of perioperative chemotherapy in non-metastatic contemporary UTUC patients using a large national database and including patients of all ages, including those younger than 65. Moreover, we aimed to ascertain the predictors of chemotherapy utilization, cancer specific mortality (CSM) and other cause mortality (OCM). One of our goals was to determine whether mortality outcomes were affected by chemotherapy exposure among all patients in general, and specifically among younger (<65) and/or high risk patients.

## RESULTS

### Clinical characteristics

Table 1 presents the demographics of the entire cohort. Of the 8,762 patients, 1402 (16%) received chemotherapy, of which 495 (35.3%) were younger than 65. Chemotherapy treated patients were younger, more likely to be married, had a larger tumor size, more aggressive stage and grade of disease, and were more likely to be treated with perioperative radiotherapy (p<0.001 for all analyses). Only 35% of the patients with high-risk disease underwent chemotherapy. Median follow-up time was 22 months (range 7-63) for both groups (p=0.73), but a higher proportion of patients treated with chemotherapy died from their disease (298 patients constituting 21.3% vs. 962 patients constituting 13.1%, p<0.001). 30.4% of the chemotherapy treated patients vs. 34.3% of the non-treated patients died from other causes, p<0.001. In patients under the age of 65, CSM was 20.4% vs. 8% (p<0.001) and OCM was 24.2%% vs. 18.3% (p<0.001) in the treated vs. non-treated group, respectively.

**Table 1:** Patient Demographics

	Chemotherapy	No Chemotherapy	p-value
**Number of patients (%)**	1402 (16%)	7360 (84%)	
**Mean Age (SD)**	67.5 (10.3)	73 (10.6)	**<0.001**
**Patients < 65 years of age (%)**	495 (35.3%)	1533 (20.8%)	**<0.001**
**Race (%)**			0363
**White**	81.8%	82.4%	
**Black**	4.9%	4.1%	
**Other**	13.3%	13.5%	
**Gender (%)**			0.105
**Male**	62.3%	40%	
**Female**	37.7%	60%	
**Marital Status (%)**			**<0.001**
**Single**	108 (8%)	615 (8.7%)	
**Married**	976 (72.7%)	4302 (61%)	
**Widowed/Divorced**	268 (19.8%)	2133 (30.3%)	
**Region (%)**			**<0.001**
**Northeast**	246 (17.5%)	1399 (19%)	
**Midwest**	217 (15.5%)	749 (10.2%)	
**South**	378 (27%)	1897 (25.8%)	
**West**	561 (40%)	3315 (45%)	
**Insured (%)**	1009 (98.3%)	4970 (98.6%)	0.478
**Mean tumor size (cm) (SD)**	4.4 (2.6)	3.85 (2.3)	**<0.001**
**Laterality (%)**			0.2
**Right**			
**Left**	48.3%	49.4%	
**Bilateral**	51.5%	50.6%	
**Location of tumor (%)**	0.2%	-	0.55
**Renal pelvis**	60.2%	61%	
**Ureter**	39.8%	39%	
**Perioperative Radiotherapy**	192 (13.8%)	146 (2%)	**<0.001**
**Pathologic Grade (%)**			**<0.001**
** Well differentiated**	2%	6%	
**Moderately differentiated**	7.3%	19%	
**Poorly differentiated**	33.3%	30.8%	
**Undifferentiated/anaplastic**	57.4%	44.2%	
**T stage (%)**			**<0.001**
**T1**	12.9%	39.3%	
**T2**	12.6%	20.9%	
**T3**	57.8%	33.1%	
**T4**	15.8%	6%	
**TX**	0.9%	0.7%	
**N stage (%)**			**<0.001**
**N0**	71.7%	94.1%	
**N1**	16%	3.6%	
**N2**	11.3%	2.1%	
**N3**	1%	0.1%	
**Num. of Patients who developed bladder cancer either before or after UTUC diagnosis, n (%)**	288 (20.5%)	1892 (25.7%)	**<0.001**
**Number of primary tumors (SD)**	1.6 (0.75)	1.7 (0.81)	**<0.001**

### Chemotherapy utilization

**Figure 1:**
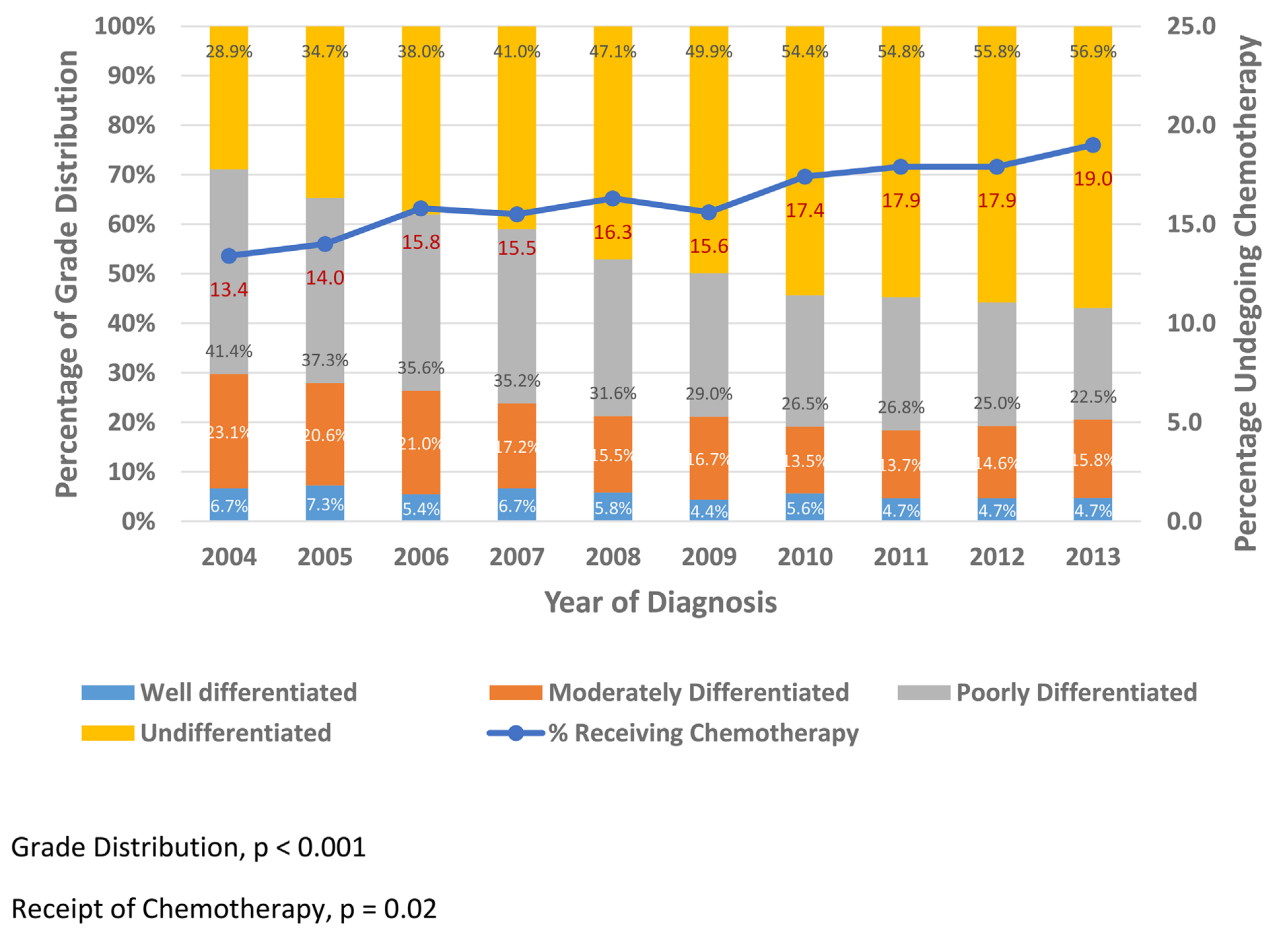
Grade Distribution and Chemotherapy Trends by Year among UTUC patients

Figure 1 shows that, through the years, the utilization of chemotherapy increased steadily (p=0.02), correlating with an increasing prevalence of higher grade disease (p<0.001).

When assessing predictors of chemotherapy utilization in patients with UTUC (Table 2), older age and residence in western states (vs. Northeast), reduced the likelihood of receiving chemotherapy. On the other hand, residence in Midwest states (vs. Northeast), tumor located in the ureter (vs. renal pelvis), worse stage (T4 vs T1) and grade disease (Undifferentiated vs Well Differentiated), and being treated with radiotherapy increased the likelihood of undergoing chemotherapy.

**Table 2:** Multivariable logistic regression analysis predicting usage of chemotherapy among UTUC patients

	p Value	OR	95.0% CI for OR	
			Lower	Upper
Gender (Female - reference)	REFERENCE			
Male	0.876	1.013	.859	1.195
Age at diagnosis	**<0.001**	**0.944**	**0.937**	**0.951**
Race (White - reference)	REFERENCE			
Black	0.726	0.935	0.64	1.365
Other	0.319	0.882	0.688	1.13
USA region (Northeast - reference)	REFERENCE			
Midwest	**0.017**	**1.423**	**1.066**	**1.9**
South	0.317	0.893	0.698	1.144
West	**0.037**	**0.779**	**0.616**	**0.985**
Insurance (No insurance - reference)	REFERENCE			
Insured	0.288	1.464	0.725	2.958
Primary upper tract site (Renal pelvis -Reference)	REFERENCE			
Ureter Location	**0.001**	**1.341**	**1.131**	**1.589**
Perioperative Radiotherapy (no therapy - reference)	REFERENCE			
Receiving radiotherapy	**<0.001**	**4.348**	**3.184**	**5.938**
T stage (T1 - Reference)	REFERENCE			
T2	**0.001**	**1.633**	**1.233**	**2.163**
T3	**<0.001**	**3.931**	**3.124**	**4.946**
T4	**<0.001**	**4.530**	**3.299**	**6.221**
N stage (N0 - Reference)	REFERENCE			
N1	**<0.001**	**3.757**	**2.884**	**4.894**
N2	**<0.001**	**4.742**	**3.481**	**6.458**
N3	**0.011**	**4.142**	**1.387**	**12.367**
Grade (Well Differentiated - Reference)	REFERENCE			
Moderately Differentiated	0.855	0.949	0.539	1.669
Poorly Differentiated	0.135	1.485	0.884	2.494
Undifferentiated	**0.011**	**1.932**	**1.162**	**3.213**

A subset analysis, specifically for patients with at least 6 months of follow-up after surgery, was performed to overcome the immortal time bias of including patients that died from their disease soon after surgery, or had any complications, precluding them of receiving adjuvant chemotherapy. Even in this subset, the same factors were predictive of chemotherapy utilization without any difference (Table 3).

**Table 3:** Multivariable logistic regression analysis predicting usage of chemotherapy among UTUC patients with at least 6 months of follow-up

	p Value	HR	95.0% CI for HR	
			Lower	Upper
Gender (Female - reference)	REFERENCE			
Male	0.953	0.994	.825	1.199
Age at diagnosis	**<0.0001**	**0.944**	**0.936**	**0.952**
Race (White - reference)	REFERENCE			
Black	0.854	0.96	0.623	1.480
Other	0.392	0.886	0.671	1.169
USA region (Northeast - reference)	REFERENCE			
Midwest	**0.029**	**1.431**	**1.037**	**1.977**
South	0.64	0.934	0.701	1.244
West	**0.049**	**0.761**	**0.58**	**0.999**
Insurance (No insurance - reference)	REFERENCE			
Insured	0.356	1.473	0.647	3.354
Primary upper tract site (Renal pelvis -Reference)	REFERENCE			
Ureter Location	**0.002**	**1.365**	**1.125**	**1.656**
Perioperative Radiotherapy (no therapy - reference)	REFERENCE			
Receiving radiotherapy	**<0.001**	**4.62**	**3.271**	**6.524**
T stage (T1 - Reference)	REFERENCE			
T2	**0.001**	**1.716**	**1.25**	**2.356**
T3	**<0.0001**	**3.835**	**2.952**	**4.983**
T4	**<0.0001**	**4.82**	**3.373**	6.889
N stage (N0 - Reference)	REFERENCE			
N1	**<0.0001**	**4.303**	**3.16**4	**5.851**
N2	**<0.0001**	**4.938**	**3.509**	**6.95**
N3	**0.033**	**3.855**	**1.118**	**13.289**
Grade (Well Differentiated - Reference)	REFERENCE			
Moderately Differentiated	0.942	1.025	0.521	2.017
Poorly Differentiated	0.195	1.514	0.808	2.835
Undifferentiated	**0.022**	**2.047**	**1.107**	**3.788**

### Predictors of cancer specific mortality

In the model identifying predictors of CSM (Table 4), older age, residence in Southern states (vs. Northeast), receipt of chemotherapy, higher stage (T4 vs T1) and higher grade disease (Undifferentiated vs Well Differentiated) increased CSM. On the contrary, more recent diagnosis, residence in Midwest states (vs. Northeast) and tumor located in the ureter (vs. renal pelvis), was protective for CSM. Similar to the analysis for the entire cohort, in patients younger than 65, receipt of chemotherapy and higher stage disease (T4 vs T1) increased CSM, while more recent disease diagnosis was protective for CSM.

**Table 4:** Fine and Gray Competing risk analysis predicting Cancer Specific Mortality among all patients and patients younger than 65

	All Patients				Patients Younger than 65			
	p Value	HR	95.0% CI for HR		p Value	HR	95.0% CI for HR	
			Lower	Upper			Lower	Upper
**Gender (Female - reference)**	REFERENCE				REFERENCE			
**Male**	0.235	1.064	0.960	1.180	0.458	1.108	0.845	1.454
**Age at diagnosis**	**<0.0001**	**1.022**	**1.016**	**1.027**	0.797	0.997	0.976	1.019
**Race (White - reference)**	REFERENCE				REFERENCE			
**Black**	0.226	1.160	0.911	1.480	.568	0.860	0.514	1.441
**Other**	0.584	0.958	0.822	1.117	0.179	1.264	0.898	1.779
**USA region (Northeast - reference)**	REFERENCE				REFERENCE			
**Midwest**	**0.043**	**0.815**	**0.667**	**0.997**	0.383	0.784	0.455	1.354
**South**	**0.021**	**1.222**	**1.030**	**1.451**	0.261	1.310	0.818	2.097
**West**	0.087	1.150	0.979	1.349	.305	1.261	0.809	1.966
**Year of diagnosis**	**<0.0001**	**0.903**	**0.887**	**0.920**	**<0.0001**	**0.882**	**0.840**	**0.925**
**Primary upper tract site (Renal pelvis -Reference)**	REFERENCE				REFERENCE			
**Ureter Location**	**0.003**	**0.845**	**0.755**	**0.946**	0.344	0.864	0.638	1.170
**Chemo therapy (no therapy - reference)**	REFERENCE				REFERENCE			
**Receiving chemotherapy**	**0.044**	**1.151**	**1.003**	**1.320**	0.038	1.380	1.018	1.871
**T stage (T1 - Reference)**	REFERENCE				REFERENCE			
**T2**	**.005**	**1.284**	**1.075**	**1.534**	**.008**	**1.869**	**1.176**	**2.972**
**T3**	**<0.0001**	**1.998**	**1.732**	**2.305**	**<0.0001**	**2.565**	**1.715**	**3.837**
**T4**	**<0.0001**	**2.932**	**2.447**	**3.510**	**<0.0001**	**4.643**	**2.814**	**7.659**
**N stage (N0 - Reference)**	REFERENCE				REFERENCE			
**N1**	**<0.0001**	**1.724**	**1.475**	**2.016**	**0.040**	**1.486**	**1.018**	**2.168**
**N2**	**0.004**	**1.458**	1.182	**1.799**	**0.020**	**1.796**	**1.089**	**2.962**
**N3**	**0.006**	**2.447**	**1.473**	**4.067**	**0.001**	**3.201**	**1.590**	**6.446**
**Grade (Well Differentiated - Reference)**	REFERENCE				REFERENCE			
**Moderately Differentiated**	0.310	1.260	0.805	1.977	0.823	1.131	0.384	3.337
**Poorly Differentiated**	**<0.001**	**2.182**	**1.431**	**3.330**	0.118	2.224	0.813	6.194
**Undifferentiate**d	**<0.001**	**2.206**	**1.449**	**3.357**	0.082	2.448	0.891	6.725

### Predictors of other cause mortality

Multivariable Cox proportional hazards model was performed to elucidate the factors predicting OCM (Table 5). Male gender, older age, black race (vs. white), tumor located in the ureter (vs. renal pelvis), and higher stage disease (T4 vs T1) all predicted an increase in OCM. However, race other than black or white (vs. white), residing in the Western or Midwestern states (vs. Northeast), and disease diagnosed more recently all reduced OCM. A subset analysis predicting OCM in patients younger than 65 (Table 5) demonstrated very similar results: older age and higher stage predicted increased OCM, while residing in the Midwest and being diagnosed at an earlier year reduced risk of OCM. However, in patients younger than 65, receipt of chemotherapy was found to increase OCM.

**Table 5:** Multivariable Cox proportional hazard model predicting Other Cause Mortality among all UTUC patients and patients younger than 65

	All Patients				Patients Younger than 65			
	p Value	HR	95.0% CI for HR		p Value	HR	95.0% CI for HR	
			Lower	Upper			Lower	Upper
**Gender (Female - reference)**	REFERENCE				REFERENCE			
**Male**	**0.006**	**1.091**	**1.025**	**1.161**	0.625	0.959	0.809	1.136
**Age at diagnosis**	**<0.0001**	**1.028**	**1.025**	**1.032**	**0.001**	**1.025**	**1.010**	**1.040**
**Race (White - reference)**	REFERENCE				REFERENCE			
**Black**	**<0.0001**	**1.312**	**1.136**	**1.515**	0.270	1.191	0.873	1.625
**Other**	**0.031**	**0.899**	**0.817**	**0.990**	0.639	0.945	0.745	1.198
**USA region (Northeast - reference)**	REFERENCE				REFERENCE			
**Midwest**	**<0.0001**	**0.541**	**0.482**	**0.607**	**<0.0001**	**0.536**	**0.384**	**0.749**
**South**	0.582	0.974	0.887	1.070	0.627	0.938	0.724	1.215
**West**	**<0.0001**	**0.820**	**0.752**	**0.896**	0.268	0.870	0.679	1.113
**Year of diagnosis**	**<0.0001**	**0.873**	**0.863**	**0.883**	**<0.0001**	**0.844**	**0.818**	**0.872**
**Primary upper tract site (Renal pelvis -Reference)**	REFERENCE				REFERENCE			
**Ureter Location**	**0.005**	**1.096**	**1.029**	**1.167**	0.324	1.093	0.916	1.303
**Chemo therapy (no therapy - reference)**	REFERENCE				REFERENCE			
**Receiving chemotherapy**	0.086	1.080	0.989	1.180	**0.027**	**1.242**	**1.025**	**1.505**
**T stage (T1 - Reference)**	REFERENCE				REFERENCE			
**T2**	**0.004**	**1.147**	**1.044**	**1.260**	**<0.0001**	**1.606**	**1.237**	**2.085**
**T3**	**<0.0001**	**1.438**	**1.328**	**1.558**	**<0.0001**	**1.837**	**1.450**	**2.328**
**T4**	**<0.0001**	**1.854**	**1.657**	**2.075**	**<0.0001**	**2.600**	**1.879**	**3.597**
**N stage (N0 - Reference)**	REFERENCE				REFERENCE			
**N1**	**<0.0001**	**1.387**	**1.240**	**1.552**	**0.003**	**1.467**	**1.139**	**1.888**
**N2**	**0.008**	**1.214**	**1.051**	**1.402**	0.244	1.237	0.865	1.770
**N3**	**0.044**	**1.533**	**1.011**	**2.323**	**0.022**	**2.125**	**1.112**	**4.060**
**Grade (Well Differentiated - Reference)**	REFERENCE				REFERENCE			
**Moderately Differentiated**	**0.032**	**0.836**	**0.709**	**0.985**	0.400	0.827	0.531	1.287
**Poorly Differentiated**	0.931	1.007	0.863	1.174	0.336	1.226	0.810	1.855
**Undifferentiated**	0.934	0.994	0.854	1.156	0.516	1.145	0.761	1.723

### High-risk patients

According to the NCCN guidelines, in high risk patients (>pT2 or > pN1), adjuvant chemotherapy should be considered [18]. Therefore, a subset analysis was also performed specifically for these patients to elucidate predictors of CSM and OCM, incorporating the role of chemotherapy (Table 6). Older age, disease diagnosis in an earlier year, not residing in Midwestern states and higher stage disease were all predictors of increased CSM and OCM. Tumor located at the renal pelvis and worse grade predicted increased CSM, while being neither white nor black, predicted decreased OCM. Chemotherapy was not found to be a significant predictor of either CSM or OCM in this high-risk cohort of patients.

**Table 6:** Fine and Gray Competing risk analysis predicting Cancer Specific Mortality and Cox proportional hazard model predicting Other Cause Mortality among high-risk UTUC patients

	Competing Risk Analysis for CSM				Cox Proportional Hazards for OCM			
	p Value	HR	95.0% CI for HR		p Value	HR	95.0% CI for HR	
			Lower	Upper			Lower	Upper
**Gender (Female - reference)**	REFERENCE				REFERENCE			
**Male**	0.145	1.09	0.971	1.224	0.241	1.045	0.971	1.124
**Age at diagnosis**	**<0.001**	**1.013**	**1.007**	**1.019**	**<0.001**	**1.021**	**1.017**	**1.025**
**Race (White - reference)**	REFERENCE				REFERENCE			
**Black**	0.672	1.063	0.801	1.41	0.103	1.155	0.971	1.374
**Other**	0.174	0.888	0.748	1.054	**0.002**	**0.838**	**0.749**	**0.937**
**USA region (Northeast - reference)**	REFERENCE				REFERENCE			
**Midwest**	**0.031**	**0.781**	**0.624**	**0.978**	**<0.001**	**0.518**	**0.452**	**0.594**
**South**	**0.019**	**1.257**	**1.038**	**1.522**	0.233	0.935	0.837	1.044
**West**	0.229	1.117	0.933	1.338	**<0.001**	**0.811**	**0.731**	**0.900**
**Year of diagnosis**	**<0.001**	**0.908**	**0.889**	**0.926**	**<0.001**	**0.887**	**0.875**	**0.899**
**Primary upper tract site (Renal pelvis -Reference)**	REFERENCE				REFERENCE			
**Ureter Location**	**0.0339**	**0.871**	**0.766**	**0.99**	0.1	1.065	0.988	1.148
**Chemo therapy (no therapy - reference)**	REFERENCE				REFERENCE			
**Receiving chemotherapy**	0.356	1.07	0.927	1.234	0.873	1.008	0.917	1.107
**T stage (T1 - Reference)**	REFERENCE				REFERENCE			
**T2**	0.506	0.864	0.563	1.328	0.585	0.922	0.687	1.235
**T3**	0.138	1.364	0.905	2.056	0.34	1.149	0.864	1.526
**T4**	**0.000**7	**2.048**	**1.352**	**3.103**	**.007**	**1.495**	**1.118**	**1.999**
**N stage (N0 - Reference)**	REFERENCE				REFERENCE			
**N1**	**<0.001**	**1.659**	**1.4**	**1.965**	**<0.001**	**1.346**	**1.195**	**1.516**
**N2**	**0.0019**	**1.4**	**1.132**	**1.73**	**0.028**	**1.180**	**1.018**	**1.367**
**N3**	**0.0005**	**2.384**	**1.458**	**3.896**	**0.048**	**1.521**	**1.003**	**2.305**
**Grade (Well Differentiated - Reference)**	REFERENCE				REFERENCE			
**Moderately Differentiated**	0.827	1.074	0.566	2.036	0.785	0.961	0.722	1.279
**Poorly Differentiated**	**0.023**	**2.004**	**1.099**	**3.655**	0.147	1.219	0.933	1.593
**Undifferentiated**	**0.035**	**1.901**	**1.045**	**3.457**	0.284	1.156	0.886	1.509

**Figure 2:**
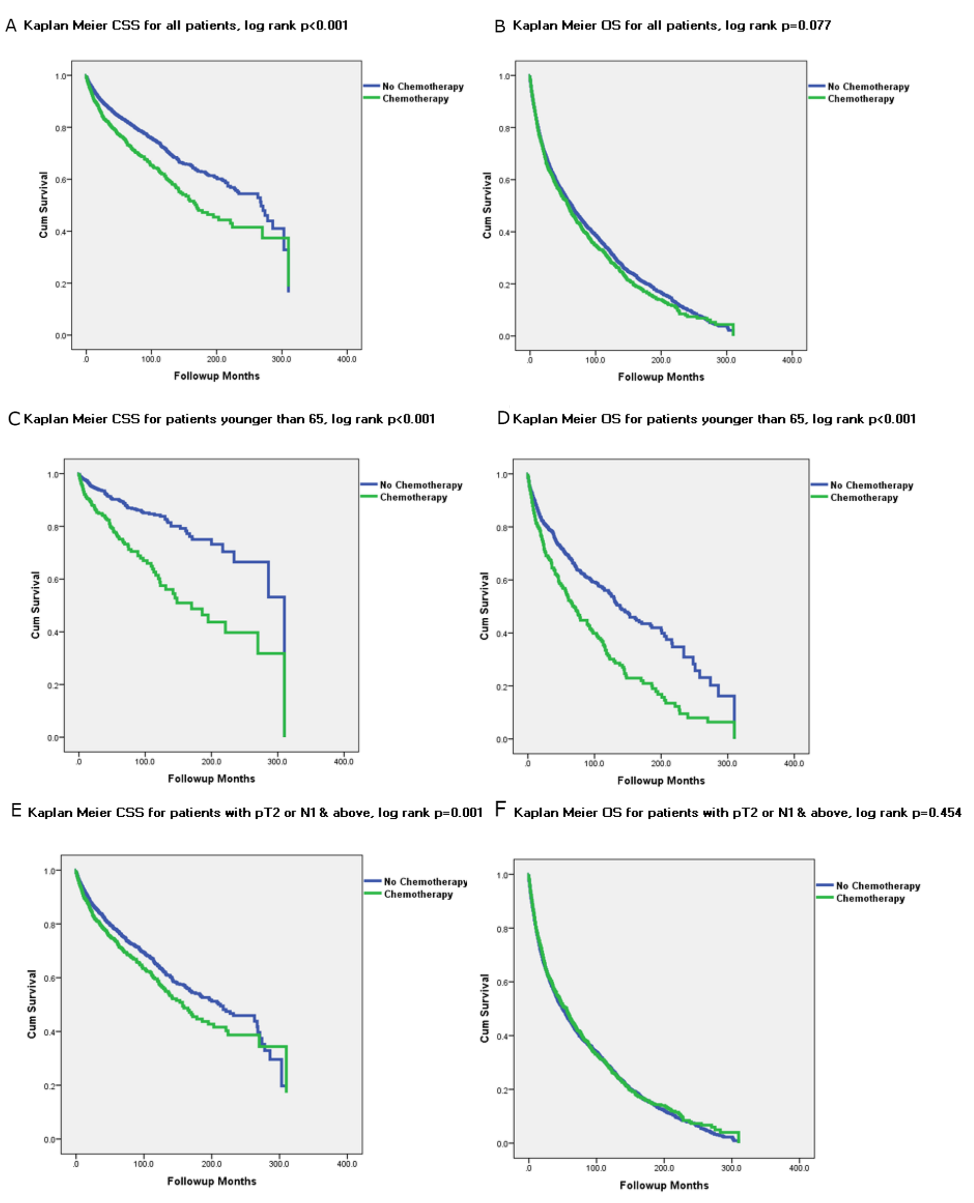
Kaplan Meier Graphs depicting Cancer Specific Survival (CSS) and Overall Survival (OS) for all subset of UTUC patients treated and not treated with chemotherapy

Figure 2 depicts Kaplan Meier (KM) graphs for all subsets of patients stratified by the receipt of chemotherapy. All KM graphs demonstrated that chemotherapy treated patients had worse CSS and either worse or similar OS.

## DISCUSSION

Our study demonstrates that approximately 16% of non-metastatic UTUC patients receive chemotherapy. Its utilization has been rising steadily in the last decade, in accordance with the increasing incidence of higher grade disease. Patients undergoing chemotherapy had higher stage and grade disease, likely accounting for higher CSM and OCM. In patients younger than 65 years old, CSM and OCM were even higher in the chemotherapy group, suggesting younger patients receiving chemotherapy had worse pathology. While perioperative chemotherapy receipt was found to be correlated to increased CSM, it was not shown to be a significantly correlated to increased OCM. Looking specifically at high-risk patients who would potentially benefit from chemotherapy, it was not found to be a significant predictor of either CSM or OCM.

Two groups have recently published their results on a large cohort of patients from the National Cancer Database. Both studies showed that predictors for chemotherapy utilization were younger age, tumor located in the ureter and higher stage and grade, [19, 20] corroborating our results. Furthermore, Cohen et al. have recently published their findings regarding usage of perioperative chemotherapy in a large cohort from the SEER-Medicare database, showing a very similar OCM in chemotherapy treated patients [17]. Although their analyses were restricted to patients older than 65, they also demonstrated that perioperative chemotherapy was demonstrated not to affect OCM and worsen CSM [17]. A large number of additional studies have also shown that perioperative chemotherapy predicts higher CSM and either worse or have no effect on OCM [21–25]. However, in contradiction to our findings, several other studies have showed an improved OS and CSS with chemotherapy, [16, 26, 27] albeit only in high-risk patients (pT3+) and in small retrospective series. Leow et al. published a meta-analysis on perioperative chemotherapy in UTUC patients, [14] and although the authors concluded that there appears to be an OS and CSS benefit for adjuvant chemotherapy, they also noted that the retrospective nature of the studies and their small sample sizes limited the strength of the evidence substantially [14]. However, Seisen et al. recently published their findings on chemotherapy utilization in a large cohort of high-risk UTUC patients (3253 patients) from the National Cancer Database, and they demonstrated a significant OS advantage for high-risk patients undergoing chemotherapy [19].

The reasons for the contradicting results in the literature regarding perioperative chemotherapy benefit in non-metastatic UTUC patients are speculative at best. Small, underpowered sample sizes and selection bias can potentially explain at least some of the inconsistent results. Moreover, the non-standardized use of chemotherapy for high-risk UTUC patients could also account for the lack of consistency in the published literature. In our study population, only 35% of high-risk patients received chemotherapy. One possible reason for the inconsistent usage could be due to the nephrotoxicity caused by cisplatin. Oncologists may refrain from starting chemotherapy in patients with a solitary kidney after a nephroureterectomy. These patients already have reduced OS due to their aggressive disease, and adding chemotherapy, with its associated toxicity to these patients with an initial renal insult, might result in a further reduction in their CSS and OS as well as impact their renal function [25]. Another reason for the contradicting results may be the lack of standardization of type, dosage and duration of chemotherapy in the different studies, potentially impacting CSS and OS.

Most of the predictors of chemotherapy utilization in our data can be intuitively explained. These include younger age, having a disease with higher stage and grade, and being treated with radiotherapy as an adjunct to surgery, due to a more aggressive disease. Tumors located at the ureter were also found to predict chemotherapy administration. This is due to the assumption that ureteral tumors were considered to have worse outcomes than renal pelvic tumors [28–30]. The proposed logic for this assumption was that the renal parenchyma played a protective role, preventing tumor invasion and progression to T3 disease and beyond. However, the issue of whether renal pelvic or ureteral tumors result in worse outcomes in UTUC has been controversial for some time [30]. There have been contradicting studies showing ureteral tumors [28, 29] and renal pelvis tumors [31] having worse outcomes.

Predictors of increased CSM in non-metastatic UTUC patients not surprisingly included older age, being diagnosed in an earlier year, and higher stage and grade disease. Interestingly, patients residing in the southern US were found to be at significantly higher risk for CSM than those residing in the Northeastern states. While we can only speculate on the reason for this, Jemal et al. have shown regional variation in prostate cancer mortality with a correlation to healthcare access [32]. They also demonstrated that prostate cancer death rates were higher in non-metropolitan compared to metropolitan regions [32]. Moreover, Liff et al. had demonstrated that patients living in rural, nonurban areas had worse disease at presentation due to poorer access care [33]. We can carefully extrapolate from these studies and surmise that UTUC patients from the southern states presented at a higher stage and grade, and therefore had increased CSM. An additional predictor of CSM was tumor located at the renal pelvis, supporting the published evidence regarding worse outcomes for this tumor location [31]. Chemotherapy, which is usually given to patients with a more advanced disease, was also demonstrated as a predictor of increased CSM. One possible explanation for this is that its benefit was not substantial enough to overcome the poor features of the aggressive disease. Supporting this was the fact that the odds ratio for higher stage and grade were considerably greater than for chemotherapy receipt, indicating that they drove the mortality more than chemotherapy receipt itself.

In a similar manner, most of the results of the model predicting OCM can also be intuitively explained. These include male gender, older age, diagnosis at an earlier year, and worse stage. Of special note, ureteral tumors were demonstrated to increase the chance of a patient dying from other causes. This is in accordance with renal pelvic tumors being shown in our data and other studies to increase CSM [31]. Interestingly, chemotherapy was shown to increase OCM only in patients younger than 65, suggesting that younger patients who underwent chemotherapy had either worse disease than the older population, or that the toxicity of chemotherapy was more substantial in the younger population. However, the results of the subset analysis of high-risk patients demonstrated no benefit from chemotherapy receipt.

The limitations of our study include its retrospective design and use of administrative datasets that lack in detailed specificity regarding a number of covariates. As in all large administrative datasets studies, there is a clear selection bias of the patients included in this study. Residual unmeasured confounding may have impacted some or all of the outcomes presented in this cohort. Without a doubt, patients receiving chemotherapy had worse disease as was shown in our study. We attempted to take this into account using multivariable analyses and, sensitivity analyses for high risk patients only. We also lacked data on the renal function of these patients, which might have precluded a significant proportion of them from receiving chemotherapy, and this was could not be taken into account in the models. Additionally, we lacked information on the protocol of chemotherapy that was given, its timing (whether neoadjuvant or adjuvant), its completion, tolerability and complications. However, Cohen’s et al. recent study, showed a 1.8% utilization of neoadjuvant chemotherapy in the last decade [17], therefore, we can carefully assume that a significant majority of our patients underwent adjuvant rather than neoadjuvant chemotherapy. Furthermore, a recent study comparing SEER treatment data with Medicare claims (limited to patients over 65) demonstrated an overall sensitivity of SEER data to identify chemotherapy in 68% of cases, while the overall positive predictive value was high (>85%) [34]. Unfortunately, information regarding the type of surgery, whether radical nephroureterectomy or a kidney sparing procedure, was mostly unavailable. There is also lack of data regarding the percentage of lymphadenectomy that was performed and the number of nodes removed. Additionally, the SEER database lacks data on comorbidities (including details on smoking), which can greatly impact the decision to give chemotherapy and OCM. Moreover, the tumor grade variable in SEER is not the same as the WHO/ISUP tumor grading system, but fortunately enough, they both correlate quite well [35]. Another important limitation is that SEER database includes data from both academic and community centers, and does not capture the surgical volume and the training levels of surgeons. Information on previous endoscopic management of ureteric or renal pelvis tumors is also not available. This could have provided additional details and enabled further data granularity for the multivariable analyses. Finally, our database includes only patients diagnosed up until 2013. It is probable, based on the trends we saw in our dataset, that CSS and OS have improved since 2013.

The strengths of our study include the fact that it is a contemporary update of a population based database with a large cohort of patients, especially when considering the rarity of this disease. Moreover, as opposed to previous study using SEER-Medicare [17], we have also reported data on patients younger than 65, giving a more extensive description of the effect perioperative chemotherapy has on disease outcomes in patients of all ages. This study exhibits a real world multicenter experience and, despite its limitations, represents contemporary evidence regarding utilization and outcomes of perioperative chemotherapy in all UTUC patients. Our data also add to the growing body of knowledge of UTUC by showing that chemotherapy predicts worse CSM and OCM for younger patients, while unfortunately having no beneficial effect on CSM and OCM in high risk patients.

In conclusion, in this large contemporary UTUC cohort, including patients of all ages and stages, chemotherapy was found to be utilized quite infrequently, but its utilization is increasing steadily. Moreover, it was found to predict higher CSM, while mostly not affecting OCM, regardless if they were high-risk or younger than 65. Despite the rarity of this disease, multicenter prospective randomized controlled trials with standardized chemotherapy protocols should be performed to determine the true effect of perioperative chemotherapy.

## MATERIALS AND METHODS

### Data collection

All patients aged 18 and older diagnosed with UTUC (International classification of diseases for oncology-2, C65.9 and C66.9 codes) between 1988 and 2013 were identified within the Surveillance, Epidemiology and End Results (SEER) cancer registry. The SEER database reports cancer specific outcomes from various geographic areas, representing 28% of the US population [36]. Demographic variables collected included age at diagnosis, gender, race, marital status, geographic location, year of diagnosis and insurance status. Clinical factors of interest included mean tumor size, tumor laterality and location (renal pelvis or ureter), use of perioperative radiation, chemotherapy, or surgical intervention (nephroureterectomy or ureterectomy), and median follow-up time. Pathological staging was based on the TNM staging system (6[th] edition – 2002) and tumor grade was defined as 1 (well differentiated), 2 (moderately differentiated), 3 (poorly differentiated) and 4 (undifferentiated), consistent with SEER grading in its most recent update.

### Patient cohort

The initial cohort included 20,407 patients. However, to determine the efficacy of chemotherapy in a cohort of strictly non-metastatic patients, patients coded as Mx or M1 were eliminated from our analysis. Additionally, we excluded 942 patients who were not operated at all, and we were left with 8,762 patients diagnosed between 2004 and 2013. Analyzed patients were divided into two groups: those who received chemotherapy versus those that did not receive chemotherapy.

### Statistical analysis

We utilized descriptive analyses (mean with standard deviation) for continuous variables, proportions for discrete variables, and comparative tests (chi-square test for discrete variables and Kruskal–Wallis test for continuous variables) in this study. Kaplan-Meier analysis (log rank test) was used to evaluate OS and CSS based on chemotherapy status in both groups. Multivariable logistic regression analyses were performed to identify factors predicting utilization of chemotherapy. Fine and Gray competing risk proportional hazards regressions were used to ascertain independent predictors of CSM and multivariable Cox proportional hazards analyses were implemented to identify predictors of OCM. Covariates in all models included gender, age, race (white, black or other), US geographic location (Northeast, Midwest, South, or West), year of diagnosis (continuous), tumor location (renal pelvis or ureter), tumor stage (pT-stage and pN-stage) and grade, and use of perioperative chemotherapy. Subset analyses for patients younger than 65 and for high-risk patients (>pT2 or pN1) were also performed to ascertain their specific predictors of CSM and OCM. Statistical tests were two-tailed and a p-value <0.05 was considered statistically significant. All analyses were conducted using the SPSS software version 23.0 (SPSS Inc., Chicago, IL) and SAS 9.4 (SAS Institute, Cary, North Carolina).

## Abbreviations

CSS: cancer specific survival
CSM: cancer specific mortality
KM: Kaplan Meier
OCM: other cause mortality
OS: overall survival
SEER: Surveillance, Epidemiology and End Results
UC: urothelial carcinoma
UTUC: upper tract urothelial carcinoma
